# EHMTI-0329. Cluster headaches - experience from a tertiary children's headache clinic

**DOI:** 10.1186/1129-2377-15-S1-B26

**Published:** 2014-09-18

**Authors:** P Prabhakar, A Gerth, C Toolis, P Goadsby

**Affiliations:** 1 Neurology, Great Ormond Street Hospital, London, UK

## Introduction

Tension Type Headache (TTH 39%) and Migraine (with and without aura 33%) are the most common primary headache seen in paediatric headache clinics. Rare headaches including Cluster Headaches (CH ) account for 8% (Abu Arafeh 2013).

## Aims

To study the prevalence and clinical characteristics of children with CH.

## Methods

A retrospective case-note study from January 2006 – December 2013 was undertaken.

## Results

Of the 472 new referrals 9 had CH (1.9 %). Mean age at presentation was 10 years 11 months (6y5m to 16y10m). Two had chronic CH and seven episodic CH. 3/9 had multiple headache types (cluster + migraine.) One had a family history of CH.

All headaches were described as severe or very severe and descriptive terms ranged from sharp to stabbing to throbbing to pounding. Location was always unilateral with neither side dominating. 7 experienced autonomic symptoms with no particular one standing out. All were agitated. 7 had some degree of photo or phonophobia. None were affected by movement.

All had normal neuroimaging (usually MRI) except one (cerebellar-pontine angle dermoid). Three had normal pituitary tests. Examination elicited occipital nerve tenderness in 7 but was otherwise normal. Mean time to diagnosis was 25 months (range 4-91). No treatment was consistently effective. Inranasal sumatriptan and occipital nerve injection were effective in three cases each.

## Conclusions

Cluster headaches can evolve over time in children. Agitation is a key feature of the condition.

No conflict of interest.

